# The benefits and future potential of generative artificial intelligence (GAI) on mental health: a Delphi study

**DOI:** 10.1080/17482631.2026.2621802

**Published:** 2026-01-27

**Authors:** Chit Thet Lal Oo, Walton Wider, Nicholas Tze Ping Pang, Eugene Boon Yau Koh, Rajkumar Krishnan Vasanthi, Khine Zar Zar Thet, Rodrigo Ramalho, Bilge Nur Özdemir, Kashmine Mahboob

**Affiliations:** a Faculty of Business and Communications, INTI International University, Nilai, Malaysia; b Department of Medical Science, University of Medicine, Mandalay, Myanmar; c Department of Applied Economic Sciences, Wekerle Sándor Üzleti Főiskola, Budapest, Hungary; d Faculty of Management, Shinawatra University, Pathum Thani, Sam Khok, Thailand; e Department of Economics, Management, and Law, International Institute of Management and Business, Minsk, Belarus; f Faculty of Medicine & Health Sciences, Universiti Malaysia Sabah, Kota Kinabalu, Malaysia; g Department of Psychiatry, Universiti Putra Malaysia, Serdang, Malaysia; h Faculty of Health and Life Sciences, INTI International University, Nilai, Malaysia; i Faculty of Medical and Health Sciences, University of Auckland, Auckland, New Zealand; j Emergency Medicine Department, Atatürk State Hospital, Antalya, Turkey; k Department of Psychiatry, Ayub Teaching Hospital, Abbottabad, Pakistan

**Keywords:** Generative artificial intelligence, mental health care services, Delphi method, ethical AI integration, long-term digital health strategy

## Abstract

**Purpose:**

This study explores expert consensus on the benefits and future potential of generative artificial intelligence (GAI) in mental health care, using the Technology Acceptance Model (TAM) to interpret these perceptions.

**Methods:**

A two-round Delphi study using a mixed-methods design was conducted with 15 purposively selected experts in psychiatry, clinical psychology, counselling, and digital mental health. Round 1 gathered open-ended responses that were thematically analysed to identify benefit and future-potential dimensions. In Round 2, experts ranked these dimensions, and consensus was assessed using Kendall’s coefficient of concordance.

**Results:**

Twenty-eight themes were identified across eight benefit dimensions, and 29 themes across eight future-potential dimensions. Statistically significant consensus was achieved for both benefits (W = 0.145, *p* = 0.034) and future potential (W = 0.152, *p* = 0.025). Accessibility and availability ranked as the most important current benefit, while AI as a collaborative and informative tool was prioritised for future application.

**Discussion:**

Experts perceived GAI as a transformative adjunct to mental health practice, particularly in expanding access, supporting personalised care, and augmenting professional capacity. Adoption is contingent on usability, transparency, trust, and robust ethical governance to ensure equitable and human-centred integration.

## Introduction

Artificial intelligence (AI) is increasingly utilised in various aspects of daily life, sparking global discussions on its benefits and drawbacks for human well-being (Ettman & Galea, 2023). As a result, both scientific and popular literature have begun to focus more closely on recent developments in AI (Allen & Woodnutt, 2023). In the field of healthcare, AI has been widely applied to support evaluation activities, such as algorithmic data processing, and to drive innovations in robotics, smart devices, and automated medication delivery systems (Al-Worafi et al., 2023; Woodnutt et al., 2024). A growing concern is the need to anticipate and manage the potential impact of advanced conversational AI on mental health (Ettman & Galea, 2023). The increasing presence of conversational AI has shown potential to support mental health through language-based interactions that convey empathy and understanding (Balcombe, 2023).

This study focuses on Generative Artificial Intelligence (GAI), specifically ChatGPT, a large language model that can generate text resembling human communication. GAI applications have been developed to provide counselling and emotional support, assist in early detection of symptoms, and promote psychoeducation (Liu et al., 2023; Srivastava & Srivastava, 2023). For example, AI-powered chatbots and conversational agents can deliver evidence-based relaxation techniques and therapeutic dialogues that help users manage anxiety and depression (Abd-Alrazaq et al., 2020; Sharma et al., 2023). By using natural language processing and machine learning, these tools can improve emotional regulation and user engagement in mental health self-care (Elyoseph et al., 2023; Liu et al., 2023). However, such systems should only be used as supplements to professional therapy rather than as replacements, especially for individuals with severe or complex mental health conditions and for those living in areas with limited access to qualified professionals (Blease & Torous, 2023; Essien & Asamoah, 2020; Miner et al., 2020; Patel et al., 2007; Torous & Blease, 2024).

At the same time, the mental health sector continues to experience a serious shortage of qualified professionals, with an estimated 150 million people in federally designated areas lacking adequate access to services (Weiner, 2022). In response, psychiatric professionals are increasingly supporting the integration of AI tools to enhance productivity and support both clinical and administrative work. AI-based systems have been shown to help automate documentation, assist in triage, and reduce diagnostic workloads, which allows clinicians to dedicate more time to direct patient care (Pham et al., 2022; Rollwage et al., 2024).

### Literature review

Within the field of mental health, GAI applications such as ChatGPT are receiving increasing attention for their ability to provide personalised support, facilitate therapeutic interactions, and assist in mental health assessments (Aditama et al., 2023). These tools contribute to the growing set of digital innovations designed to improve mental health outcomes and expand access to care. This review examines the impact of GAI on mental health, focusing on its benefits, limitations, and future potential in facilitating human interaction through AI-driven conversational systems.

A substantial body of research has explored the implications of AI in mental health, emphasising its capacity to enhance diagnostic accuracy, improve treatment outcomes, and extend access to care through technology-driven solutions (Elyoseph et al., 2023). For instance, OpenAI introduced the Chat Generative Pre-training Transformer (ChatGPT) on November 30, 2022, as a highly capable conversational system developed in San Francisco (Singh, 2023). Trained on extensive textual data, GAI can produce written responses that resemble human language and thought patterns (Naidu & Sevnarayan, 2023). Such capabilities suggest that these systems could play a transformative role in delivering virtual mental health care.

AI-based conversational systems have gained prominence as promising tools for psychological support and treatment (Singh, 2023). GAI, with its ability to conduct lifelike conversations, introduces a new form of self-guided online intervention for individuals experiencing symptoms of depression and anxiety (Pandya et al., 2023). The natural and user-friendly design of chatbots makes them appealing in psychotherapy, as they offer convenience, accessibility, and the ability to simulate empathetic dialogue. However, researchers have also raised concerns about potential limitations, including algorithmic bias, the risk of overreliance on AI-generated empathy, and the lack of human accountability in automated interactions (Blease & Torous, 2023; Hitch, 2023).

A key advantage of naturalistic chatbots is their accessibility (Abd-Alrazaq et al., 2020). Because they are continuously available, chatbots offer immediate and convenient access to mental health support. This is particularly valuable for individuals living in underserved regions or those facing barriers to traditional therapy due to cost, scheduling, or distance (Liu et al., 2023). Despite these benefits, concerns persist about privacy, data protection, and the reliability of algorithmic decision-making (Ziems et al., 2022).

In addition to increasing access, GAI has been identified as a potential tool for alleviating psychiatric burnout, which affects nearly half of mental health professionals (Cheng et al., 2023). By supporting administrative functions and providing structured assistance based on evidence-informed therapeutic principles, GAI can reduce workload pressures and enhance efficiency (Hamdoun et al., 2023). Furthermore, GAI systems can help reduce stigma by encouraging individuals to seek help and by normalising mental health discussions in everyday contexts (Ziems et al., 2022). Through conversational engagement, these tools may help users reframe negative thoughts and promote self-compassion, thereby supporting the normalisation of care-seeking behaviour (Sharma et al., 2023).

Recent findings underscore the growing importance of collaboration between GAI systems and mental health professionals, highlighting that these tools are most effective when used to augment, rather than replace, human expertise. Studies have argued that mental health professionals must actively shape how AI is integrated into psychiatric care to ensure that its deployment aligns with ethical, clinical, and empathic standards (Moran, 2024). Similarly, healthcare integration roadmaps recommend co-design processes involving clinicians, AI developers, and patients to promote systems that enhance trust, transparency, and clinical accuracy (Yu et al., 2023). In practical terms, clinicians may instruct patients to use GAI between therapy sessions for cognitive-behavioural journaling or thought-record exercises, use GAI within sessions for structured risk prompts or safety plan templates, and deploy GAI triage systems during intake to assist in preliminary screening before clinician review. In such settings, collaboration ensures that GAI complements professional judgement rather than substitutes for it, particularly in sensitive diagnostic and therapeutic applications (Blease & Torous, 2023).

Moreover, empirical evidence supports the potential of GAI to improve empathy and the quality of clinical communication. A large-scale systematic review and meta-analysis found that ChatGPT-generated responses to medical and health-related questions were rated significantly higher in empathy and quality than those provided by human healthcare professionals, with a standardised mean difference of 0.87 in favour of AI (Howcroft et al., 2025). Likewise, a mixed-methods study demonstrated that GPT-3.5 and GPT-4.0 models produced patient-facing clinical notes with greater prosocial and empathic language than traditional human-authored notes. However, occasional lapses in medical precision were noted (Kharko et al., 2024). These findings align with earlier work, which has shown that AI-assisted responses can enhance both accuracy and empathy in patient interactions (Tate et al., 2023).

Evidence from human–AI collaboration studies in digital mental health further demonstrates that AI feedback can improve empathy and communication quality in support interactions. For example, Sharma et al. (2023) found that introducing an AI feedback system in online peer-to-peer mental health support increased conversational empathy by nearly 20% overall and almost 40% among less-experienced supporters. This finding aligns with the broader clinical perspective that supervised GAI can act as a collaborative assistant, offering empathetic language, educational materials, and draft treatment summaries for clinicians to review and refine.

Despite these promising findings, the question of how far GAI should be integrated into mental health practice remains open. The evidence base concerning its long-term benefits and limitations is still developing (Hitch, 2023). Should robust empirical data confirm that GAI can effectively supplement or improve certain aspects of care, its use may become an essential component of modern mental health services (Elyoseph et al., 2023). Optimistically, GAI could enhance patient outcomes, deliver cost-effective interventions at scale, and help mitigate longstanding disparities in access to care.

The integration of GAI into clinical practice has also sparked ethical and professional discussions concerning its appropriate role and regulation. Scholars have called for clear guidelines to ensure that AI enhances rather than replaces human expertise and clinical judgement (Cheng et al., 2023; Doraiswamy et al., 2020). Moreover, while GAI contributes to the destigmatisation of mental health by making resources more approachable and fostering self-help behaviour (Sharma et al., 2023), it also raises critical questions about authorship, accountability, and task distribution in AI-assisted care (Hitch, 2023).

Finally, although GAI offers scalable solutions, its integration must be undertaken carefully, with close attention to ethical considerations, data governance, and environmental sustainability, given the significant computational resources required to train and maintain large language models (Allen & Woodnutt, 2023). In light of these complexities, this study investigates expert perspectives on the benefits and future potential of GAI in mental health care. Building on this literature, the following section outlines the theoretical framework that guides the study.

### Underpinning theory

The Technology Acceptance Model (TAM) provides the theoretical foundation for this study, offering a systematic framework for understanding how individuals accept and use new technologies. Developed by Davis (1989), TAM proposes that two primary constructs, Perceived Usefulness (PU) and Perceived Ease of Use (PEOU), influence an individual’s attitude toward a technology, which subsequently affects their intention to use it. Over the years, TAM has been expanded to include additional dimensions such as trust, risk perception, and ethical awareness, particularly in fields involving sensitive human interaction such as healthcare (Ko & Leem, 2021; Ratta et al., 2025).

PU refers to the degree to which individuals believe that a technology enhances their work performance or outcomes. Recent research consistently confirms that PU remains one of the strongest predictors of technology adoption across diverse domains (Ma et al., 2025). In mental health practice, demonstrating that GAI systems can enhance therapeutic communication, decision-making, and emotional support is crucial for encouraging their adoption. Evidence suggests that when clinicians and patients perceive AI systems as helpful in improving therapeutic efficiency and the quality of emotional care, their intention to adopt and continue using these tools increases significantly (Békés et al., 2025). When both professionals and patients recognise tangible benefits such as greater efficiency, improved insight, and enhanced emotional care, the perceived value and acceptance of GAI systems strengthen accordingly (Kauttonen et al., 2025).

PEOU relates to how effortless and intuitive a technology is perceived to be. Studies have shown that usability and accessibility significantly influence user trust and intention to use a system (Al-Abdullatif, 2024). In mental health contexts, simple, clear, and responsive conversational systems help create a sense of comfort and psychological safety for both clinicians and patients (Heston, 2023). Studies show that intuitive and emotionally attuned conversational AI systems, such as hybrid chatbots, enhance engagement, reduce communication anxiety, and foster a sense of therapeutic presence (Wah, 2025). Ease of interaction has also been shown to reduce anxiety around technology use and encourage sustained engagement, especially when conversational agents maintain human-like empathy and clarity (Babu & Joseph, 2024).

Trust and risk are recognised as critical extensions of the original TAM and have become increasingly central in health-related applications. Empirical studies in healthcare and AI adoption consistently find that trust mediates between user intention and acceptance, while perceived risk negatively influences acceptance (Goh et al., 2024; Kauttonen et al., 2025). Users are more likely to adopt digital systems when they believe that ethical standards, data privacy, and accountability are well established (Balaskas et al., 2025; Choudhury et al., 2025). In mental health care, where confidentiality and empathy are vital, building transparent and ethically governed AI systems is a prerequisite for clinical acceptance and long-term integration into professional practice (Saeidnia et al., 2024; Svensson et al., 2025). Recent studies emphasise that transparency, fairness, and accountability are essential for trustworthy AI systems that protect patient autonomy and privacy while maintaining clinician–patient trust (Cheong, 2024; Ott & Dabrock, 2022).

Attitude toward use reflects an individual’s overall positive or negative feeling toward using a technology and is a direct predictor of behavioural intention (Ovsyannikova et al., 2025). In the context of mental health, positive emotional experiences when interacting with AI-assisted tools can strengthen engagement and trust (Peng et al., 2025). Users who perceive AI systems as empathetic, reliable, and beneficial are significantly more willing to continue using them (Shen et al., 2024).

By applying TAM and its extended constructs, this study aims to understand how perceived usefulness, ease of use, trust, and attitudes influence the acceptance and implementation of GAI in mental healthcare. Using the Delphi method, expert insights were gathered to identify key opportunities, challenges, and ethical considerations shaping the future integration of generative technologies into mental health practice.

## Methods

### Research design

To achieve a comprehensive understanding of expert perspectives, this study adopted a mixed-methods design combining qualitative and quantitative approaches. A two-round Delphi technique was employed to facilitate iterative data collection and analysis (Tee et al., 2022). This qualitative consensus-based method enabled a panel of experts to systematically evaluate and refine their views through successive rounds of feedback.

Fifteen experts with professional backgrounds in both technology and mental health participated, contributing diverse and specialised insights into the role of GAI in mental health practice (Niederberger & Spranger, 2020). Thematic analysis was employed to identify key themes and recurring patterns within the open-ended responses. To statistically assess the level of agreement among participants across Delphi rounds, Kendall’s coefficient of concordance (W) was applied.

The integration of qualitative interpretation and quantitative validation strengthened the reliability and depth of the study findings, ensuring a well-rounded understanding of expert consensus regarding the benefits and future potential of GAI in mental health care.

### Sampling

Fifteen mental health professionals were selected through purposive sampling to ensure a well-informed and contextually relevant expert panel (Beiderbeck et al., 2021). All participants were individuals with whom the authors have established professional collaborations through academic, clinical, and research networks. The experts were drawn from psychiatry, clinical psychology, counselling, and mental health technology, representing both academic and clinical practice domains.

Selection was guided by specific inclusion criteria: a minimum of five years of professional experience in mental health practice, active involvement in digital or AI-related mental health initiatives. It demonstrated scholarly or clinical contributions to the field. Potential participants were identified through university affiliations, hospital partnerships, and collaborative research projects in which the authors were involved.

Personalised email invitations were sent to each expert, outlining the study objectives, participation criteria, and ethical assurances of confidentiality and voluntary engagement. The final panel consisted of psychiatrists, counsellors, clinical lecturers, medical doctors, and researchers, all of whom actively contribute to mental health education, clinical supervision, and technological innovation in practice. This multidisciplinary composition ensured that the Delphi process captured a balanced synthesis of academic insight and practical experience, thereby enhancing the credibility and depth of the findings.

### Instruments and pre-test

The first-round questionnaire consisted of two open-ended questions:

1. List how GAI will be beneficial for mental health.
2. List potential uses of future GAI in mental health.

To ensure clarity and relevance, a pre-test was conducted with two psychology experts who reviewed the wording and provided feedback to refine the items. This pilot step ensured that the questions were framed appropriately to elicit meaningful and comprehensive responses from the expert panel.

Experts received the first-round questionnaire via email and provided written responses within the designated timeframe. These written submissions were compiled, transcribed, and reviewed in preparation for analysis. As all inputs were provided in written form, the statements used in the manuscript, including illustrative quotations, reflect the experts’ original phrasing, with only minor typographical adjustments made for readability and consistency. The responses were then analysed using thematic analysis to identify recurring concepts, patterns, and unique insights. This analytical process enabled categorising expert inputs into coherent thematic dimensions, which subsequently informed the structure of the second-round questionnaire (Deveci et al., 2020).

Insights from the first round were refined during the second-round Delphi process (Parker et al., 2021). A synthesised list of themes generated from Round 1 served as the basis for the second-round instrument, in which experts were asked to rank the importance of each dimension based on their experience and judgement (Humphrey-Murto et al., 2020). The ranking procedure required experts to assign unique numerical values to each item, ensuring clarity, eliminating duplicate rankings, and enhancing statistical validity (Parker et al., 2021).

A structured ranking questionnaire facilitated systematic evaluation and yielded a prioritised list of both current benefits and anticipated future roles of GAI in mental health. Although attrition is often expected in Delphi studies (Niederberger & Spranger, 2020), participation remained consistent across both rounds. The two rounds were completed within a one-month period, ensuring timely and efficient data collection and analysis.

### Data analysis

This study employed thematic analysis to systematically and credibly capture and interpret expert perspectives (Naisola-Ruiter, 2022). The analytical process followed Braun and Clarke (2006) six-phase approach, which involved becoming familiar with the data, generating initial codes, searching for themes, reviewing themes, defining and naming themes, and producing the final report.

The thematic analysis was guided by the TAM, which provided the theoretical foundation for examining expert insights into the use of GAI in mental health care. The key constructs of TAM, namely perceived usefulness, perceived ease of use, trust, and attitude toward use, were used as guiding concepts for interpreting the data. During the coding process, expert responses were examined and categorised according to these constructs to ensure that the emerging themes were theoretically grounded and linked to established principles of technology acceptance.

The first set of responses from the Delphi study was carefully examined, transcribed, and coded to identify key patterns and recurring ideas. The coding process was iterative and data-driven while remaining guided by the conceptual structure of TAM to maintain analytical consistency. Data were organised and coded manually using Microsoft Excel as a practical tool for sorting, labelling, and grouping responses (Lochmiller, 2021). Each expert statement was reviewed in detail, and descriptive codes were assigned to capture both recurring concepts and unique insights related to the potential benefits and challenges of GAI in mental health. This process resulted in the identification of several themes that reflected expert perspectives on the benefits, limitations, and ethical implications of using GAI in clinical and therapeutic settings.

The second phase of analysis employed Kendall’s W to determine the degree of agreement among experts in ranking the identified elements. Kendall’s W, a normalised form of the Friedman test statistic, is useful for assessing inter-rater reliability and consensus in Delphi studies. The coefficient ranges from 0, indicating no agreement, to 1, indicating complete agreement, with a *p*-value of less than 0.05 signifying statistical significance. In this study, the results showed consistent agreement among experts in evaluating the key benefits of GAI, confirming the reliability of the consensus. Although a lower Kendall’s W value may suggest variation in individual rankings, the *p*-value remained below 0.05, indicating sufficient agreement among participants. Therefore, a third round of the Delphi study was not required, as the consensus had been adequately achieved.

### Ethical considerations

Ethical approval for this study was obtained from the Ethics Committee of INTI International University (approval code: INTI/UEC/2024/005). All expert participants were informed of the study’s objectives, procedures, and their rights related to participation. Written informed consent was obtained from all participants before their participation. As this study involved only adult participants, no assent procedures were necessary.

## Results

### First round of the Delphi method

On March 7, 2024, invitation letters were issued via email to 15 mental health practice experts to participate in the Delphi method. This study used the Delphi approach to gather expert opinions and ideas regarding the benefits and future possibilities of GAI in mental health. All 15 provided positive responses, resulting in a 100% response rate in this study (Bucyibaruta et al., 2023). Table I presents the profiles of 15 experts who participated in the Delphi approach.

**Table I.** Experts’ profiles.

No.	Job title	Background of organisation
E1	Lecturer, Psychiatrist	Faculty of Medicine, Public University (Southeast Asia)
E2	Medical Doctor (MD, MPH)	Department of Psychiatry, Public University (Europe)
E3	Psychiatrist, Clinical Supervisor	Public University/National Health Agency (Asia)
E4	Registrar (MRCPsych)	University Hospital (Southeast Asia)
E5	Medical Lecturer, Psychiatrist	Public University (Southeast Asia)
E6	Programme Officer of Counselling	Faculty of Medicine, Private University (Asia)
E7	Clinical Lecturer	Psychiatric Department, Faculty of Medicine (Asia)
E8	Consultant Psychiatrist	National University Hospital (Asia)
E9	Registrar (DrPsych)	University Health Board (UK)
E10	Psychiatrist	Public Medical University (Middle East)
E11	Academic Secretary, Senior Researcher	National Medical Research Centre (Eastern Europe)
E12	Consultant	Specialty Hospital (South Asia)
E13	Researcher (MD, PhD)	School of Public Health, Public University (Oceania)
E14	Medical Doctor (MBBS)	Teaching Hospital (South Asia)
E15	Clinical Lecturer, Psychology	Faculty of Medicine and Health Sciences, Public University (Southeast Asia)

The first round of the Delphi study yielded a substantial body of qualitative data, reflecting expert insights into the benefits and future potential of GAI in mental health. The responses were coded and synthesised into broader interpretive patterns. This led to the development of 28 distinct themes, which were subsequently organised into eight overarching dimensions. These dimensions represent key conceptual domains, such as accessibility, personalisation, cost-efficiency, clinical support, psychoeducation, crisis intervention, data utility, and ethical considerations, each capturing a particular facet of GAI’s contribution to mental health care. The number of themes within each dimension varies based on the richness and diversity of expert perspectives. A detailed breakdown is presented in Table II.

Similarly, an analysis of expert responses concerning the anticipated future potential of GAI in mental health yielded 29 themes. These were interpreted and clustered into eight thematic dimensions encompassing areas such as diagnostic innovation, therapeutic modalities, technical limitations, professional augmentation, digital outreach, early intervention, global ethical considerations, and collaborative AI-human engagement. These forward-looking dimensions reflect how experts envision the evolution of GAI in both clinical and non-clinical mental health contexts. The full classification of the themes and dimensions is presented in Table III.

The second round of the Delphi method was based on the results of the first round. Because their expertise and experience enhanced the following rounds, we are grateful for the response from the experts who consented to participate in the first round. It was anticipated that the attendance of experts would provide valuable professional expertise and insights, significantly advancing this study (Spranger et al., 2022).

### Second round of the Delphi method

The second round of the Delphi process was conducted on March 15, 2024. The first-round responses were categorised into dimensions based on thematic analysis, as indicated in Tables II and III. The same fifteen participants who participated in the first round of the Delphi process also participated in the second round. In the second round, experts were invited to rank the dimensions based on their perceived significance. The experts were instructed to assign a rank from 1 to 8 to each dimension, with 1 indicating the most significant and 8 the least significant. They were required to assign distinct rankings without duplication to ensure clarity and to facilitate statistical analysis (Boel et al., 2021).

**Table II.** Consolidation of round one Delphi findings regarding the benefits of GAI on mental health.

No.	Dimension	Themes
1	Accessibility and Availability	Expanded reach to underserved and remote communities 24/7 availability of mental health support Reduction of social stigma through anonymous support Alleviation of geographical and mobility constraints Supplementary companionship during wait times
2	Personalisation and Prediction	Adaptive treatment planning based on user needs Early identification of mental health symptoms Emotion-sensitive intervention suggestions Predictive monitoring for stress and crisis AI-assisted diagnosis and tracking
3	Cost and Resource Efficiency	Reduced reliance on human therapists in low-risk cases Low-cost mental health intervention Optimisation of service delivery in resource-limited settings
4	Treatment and Rehabilitation	Immersive simulations for therapeutic exposure GAI-assisted therapeutic modalities (e.g., VR-based cognitive therapy) Technology-enhanced early intervention strategies
5	Education and Awareness	Delivery of psychoeducational content Promotion of mental health literacy Reinforcement of self-management and adherence
6	Support and Crisis Intervention	Immediate support during psychological crises Automated triage and escalation systems Clerical and logistic support for mental health professionals
7	Data Analysis and Research	AI-enhanced synthesis of research insights Evidence generation for mental health policymaking Pattern detection in large-scale mental health data
8	Ethical and Quality Considerations	Privacy, security, and data ethics in GAI systems Risks of over-reliance and algorithmic bias Need for guidelines and regulation in clinical AI use

**Table III.** Consolidation of round one Delphi findings regarding the future potential of GAI on mental health.

No.	Dimension	Themes
1	Diagnostic and Personalised Treatment	Precision diagnosis using multimodal data Genomic and lifestyle-informed treatment plans Algorithm-assisted medication decisions Predictive risk modeling and early detection
2	Therapeutic Tools and Modalities	Safe, judgement-free therapeutic environments AI as empathic interlocutor for emotional processing Delivery of digital cognitive-behavioural tools Simulated therapeutic scenarios (e.g., exposure) Narrative-based interventions and emotional journaling
3	Technological and Computational Limitations	Constraints of AI in modelling human consciousness Risks of technological stagnation or misuse Complexity of modelling psychological states
4	AI as Support for Professionals	Automated session documentation and summarisation Enhanced clinical decision-making support Administrative workload reduction for clinicians
5	Digital Learning and Outreach	AI-enabled special education support Mass dissemination of mental health knowledge Digital campaigns for prevention and stigma reduction
6	Affordability, Accessibility, and Early Intervention	Cost-effective delivery of services at scale Crisis-time AI intervention pathways Proactive screening and triaging capabilities Reduction of structural barriers to care
7	Global Implications and Ethical Considerations	Addressing digital divides between Global North and South Ensuring cultural sensitivity and local adaptation Accountability frameworks for ethical deployment
8	AI as a Collaborative and Informative Tool	AI as co-pilot in patient-clinician dialogue Augmented shared decision-making processes Empowerment through AI-mediated health literacy AI as a bridge for lay-public understanding

Table IV summarises the ranking results for the benefits of GAI on mental health. “Accessibility and Availability” ranked as the most significant dimension (M = 2.73), emphasising its potential to enhance access to mental health services, particularly in underserved areas. Following this, “Education and Awareness” (M = 3.73) and “Personalisation and Prediction” (M = 4.47) were ranked second and third, respectively, highlighting the ability of GAI to disseminate mental health knowledge and tailor interventions to individual needs. Other dimensions, such as “Support and Crisis Intervention” (M = 4.40) and “Data Analysis and Research” (M = 4.67), were also perceived as valuable. In contrast, “Cost and Resource Efficiency” (M = 5.07) and “Treatment and Rehabilitation” (M = 5.20) were ranked lower. “Ethical and Quality Considerations” were identified as the least significant dimensions (M = 5.73), likely reflecting concerns over the challenges of maintaining ethical standards and quality in GAI applications.

**Table IV.** Second round of the Delphi method concerning the benefits of GAI on mental health.

Experts	Dimensions							
	1	2	3	4	5	6	7	8
E1	8	1	2	3	5	6	7	4
E2	4	6	3	7	2	5	1	8
E3	1	8	6	7	5	2	3	4
E4	8	7	4	5	3	2	1	6
E5	6	1	7	5	8	4	3	2
E6	1	6	2	8	3	7	5	4
E7	1	5	6	7	4	3	2	8
E8	1	5	7	4	3	6	2	8
E9	1	4	5	6	2	7	3	8
E10	2	5	4	6	1	3	7	8
E11	1	2	5	3	7	4	8	6
E12	1	8	4	7	3	2	5	6
E13	4	2	8	1	6	5	7	3
E14	1	4	7	5	2	3	8	6
E15	1	3	6	4	2	7	8	5
Means	2.73	4.47	5.07	5.20	3.73	4.40	4.67	5.73
Group Ranking	1	4	6	7	2	3	5	8

Note: 1 = Accessibility and Availability; 2 = Personalisation and Prediction; 3 = Cost and Resource Efficiency; 4 = Treatment and Rehabilitation; 5 = Education and Awareness; 6 = Support and Crisis Intervention; 7 = Data Analysis and Research; and 8 = Ethical and Quality Considerations.

As shown in Table V, the future potential of GAI revealed that “AI as a Collaborative and Informative Tool” was ranked as the most significant dimension (M = 3.40), indicating strong support for its role in enhancing professional mental health interventions. This was followed by “AI as Support for Professionals” (M = 3.47) and “Diagnostic and Personalised Treatment” (M = 3.67), which highlight the GAI’s capacity to complement mental health professionals and provide tailored solutions. Other dimensions, such as “Digital Learning and Outreach” (M = 4.33) and “Therapeutic Tools and Modalities” (M = 4.73), also garnered recognition. In contrast, “Affordability, Accessibility, and Early Intervention” (M = 4.87) and “Global Implications and Ethical Considerations” (M = 5.73) were perceived as less critical. Finally, “Technological and Computational Limitations” were ranked as the least significant dimension for future potential (M = 5.80), reflecting lower concerns about barriers compared with other transformative applications of GAI. These rankings provide valuable insights into expert priorities, highlighting key focus areas for maximising benefits and addressing challenges associated with GAI in mental health (Boel et al., 2021).

**Table V.** Second round of the Delphi method concerning the future potential of GAI on mental health.

Experts	Dimensions							
	1	2	3	4	5	6	7	8
E1	1	2	8	3	6	7	5	4
E2	6	3	2	4	7	5	8	1
E3	8	7	6	5	4	3	1	2
E4	1	4	5	2	3	6	8	7
E5	1	5	4	2	7	6	3	8
E6	6	5	7	2	3	4	8	1
E7	4	5	3	6	7	8	1	2
E8	2	3	6	1	4	5	8	7
E9	3	4	5	1	2	6	8	7
E10	4	7	8	2	5	6	3	1
E11	1	6	7	5	2	3	8	4
E12	6	7	5	8	4	1	3	2
E13	3	6	8	2	4	5	7	1
E14	3	2	6	8	5	4	7	1
E15	6	5	7	1	2	4	8	3
Means	3.67	4.73	5.80	3.47	4.33	4.87	5.73	3.40
Group Ranking	3	5	8	2	4	6	7	1

Note: 1 = Diagnostic and Personalised Treatment; 2 = Therapeutic Tools and Modalities; 3 = Technological and Computational Limitations; 4 = AI as Support for Professionals; 5 = Digital Learning and Outreach; 6 = Affordability, Accessibility, and Early Intervention; 7 = Global Implications and Ethical Considerations; 8 = AI as a Collaborative and Informative Tool.

The level of agreement among the experts was evaluated using Kendall's W, which is a measure of consensus that ranges from 0 (no agreement) to 1 (total agreement). Kendall’s W for the benefits of GAI was 0.145 with a statistically significant *p*-value of 0.034. For the future potential of GAI, Kendall’s W was 0.152 with a *p*-value of 0.025. These results confirm a statistically significant level of consensus among experts in both rounds. Statistically significant *p*-values (<0.05) indicated that the rankings were consistent and reliable, thereby negating the need for a third round of the Delphi process.

### Synthesis of Delphi findings into thematic domains

To interpret these findings more cohesively, both the benefit and future potential dimensions were synthesised through the theoretical lens of the TAM. The four key constructs of TAM (perceived usefulness, perceived ease of use, trust, and attitude toward use) guided the consolidation process. This approach ensured that the final thematic domains not only reflected empirical consensus but were also grounded in an established framework that explained technology adoption behaviour among mental health professionals.

Overlapping or conceptually related dimensions from both datasets were merged into six overarching domains. This refinement enabled thematic clarity while preserving the richness of expert perspectives. Each domain represents a major conceptual area that captures both the practical and theoretical implications of integrating GAI into mental health practice. The consolidation process is summarised in Table VI.

**Table VI.** Integration of Delphi-derived dimensions into six thematic domains.

Final discussion domain	Merged source dimensions	Dominant TAM constructs
1. Democratising Access and Enhancing Early Intervention	Accessibility and Availability; Affordability, Accessibility, and Early Intervention	Perceived Usefulness, Perceived Ease of Use
2. Personalisation and Predictive Precision	Personalisation and Prediction; Diagnostic and Personalised Treatment	Perceived Usefulness, Trust
3. Augmenting Clinical Practice and Supporting Professional Capacity	Support for Professionals; Treatment and Rehabilitation	Perceived Usefulness, Attitude Toward Use
4. Transforming Therapeutic Modalities and Experiences	Therapeutic Tools and Modalities; AI as a Collaborative and Informative Tool	Attitude Toward Use, Trust
5. Leveraging Collective Intelligence for Research and Public Health	Data Analysis and Research; Education and Awareness; Digital Learning and Outreach	Perceived Usefulness
6. Ethical Governance and Global Accountability	Ethical and Quality Considerations; Global Ethical Implications	Trust, Attitude Toward Use

## Discussion

This Delphi study examined expert perspectives on the benefits and future potential of GAI in mental health care. Guided by the TAM, the findings illustrate how perceived usefulness, perceived ease of use, trust, and attitude toward use shape experts’ evaluations of GAI and their expectations for its integration into clinical and non-clinical settings. The six thematic domains derived from the Delphi results collectively reflect emerging priorities in digital psychiatry, centred on equity, personalisation, efficiency, and ethical governance.

### Democratising access and enhancing early intervention

Experts consistently emphasised accessibility and availability as the most transformative contributions of GAI to mental health care, aligning with the TAM construct of perceived usefulness. Participants agreed that GAI increases the perceived utility of mental health services by providing on-demand, location-independent support that addresses barriers of geography, cost, and time. As one expert shared, *“Mental health access is no longer limited by physical distance or clinic hours. GAI allows people to access support on their terms, even from places where services don’t exist”* (E3). These perspectives align with prior findings that demonstrate conversational AI and chatbot systems can expand the reach of care and promote self-help behaviours among underserved populations (Abd-Alrazaq et al., 2020; Liu et al., 2023; Sharma et al., 2023).

Experts also described GAI as useful for early detection, triage, and preventive intervention. One participant stated, *“AI is especially helpful during crises. It offers 24/7 access and supports adolescents who might never walk into a clinic because of fear or shame”* (E15). This reflects a strong belief in the usefulness of GAI for supporting low-intensity, early-stage care consistent with the stepped-care approach (Patel et al., 2007). Previous research has similarly underscored that conversational AI can deliver empathetic, language-based interactions that facilitate early symptom identification and emotional regulation (Balcombe, 2023; Ettman & Galea, 2023).

However, perceived ease of use and trust emerged as critical moderating factors influencing adoption. As one expert cautioned, *“Access means nothing if it’s not equitable. Many don’t have devices, data plans, or culturally relevant interfaces”* (E14). This reinforces that accessibility extends beyond technological availability to include usability and inclusivity. Without addressing digital inequities, culturally adaptive design, and infrastructure gaps, the perceived usefulness of GAI will not translate into sustained engagement. Experts also emphasised that the perceived trustworthiness of GAI depends on transparent data handling, ethical implementation, and responsible governance, concerns that are echoed in recent work highlighting the importance of accountability in mental-health-focused AI (Blease & Torous, 2023; Torous & Blease, 2024). Future policies should therefore prioritise equitable usability, reliability, and ethical compliance to fulfil the democratising promise of GAI (Allen & Woodnutt, 2023).

### Personalisation and predictive precision

The second major dimension identified in the Delphi results centres on personalisation, which strongly corresponds to perceived usefulness and positive attitudes toward GAI. Experts noted that GAI can adapt language, tone, and recommendations based on user responses, resulting in a more personalised therapeutic experience. As one participant explained, *“AI doesn’t just follow scripts. It learns how the user reacts and changes the strategy accordingly. This is dynamic personalisation at scale”* (E1). Such adaptability aligns with earlier findings that natural-language models, such as ChatGPT, can generate empathetic and contextually sensitive responses that enhance engagement and emotional support (Elyoseph et al., 2023; Howcroft et al., 2025). When users perceive technology as responsive and outcome-enhancing, their acceptance increases significantly.

Several experts envisioned future advancements in diagnostic precision through the integration of multimodal data, including behavioural, physiological, and environmental indicators. One expert described, *“AI-driven treatment that considers sleep, diet, emotional states, and even genetic risk factors. It’s a living care plan that updates itself”* (E3). This vision aligns with broader healthcare innovations that employ machine learning to refine prediction accuracy and personalise care pathways (Al-Worafi et al., 2023; Pham et al., 2022).

Nevertheless, personalisation raises concerns about trust and risk perception, key extensions of TAM in sensitive domains such as mental health. As one expert cautioned, *“Predicting someone’s risk of depression sounds helpful, but it could backfire. People might panic or feel doomed, especially if the message isn’t delivered sensitively”* (E9). This tension reflects ongoing debates on algorithmic bias and psychological safety in AI-mediated interventions (Doraiswamy et al., 2020; Hitch, 2023). Transparency in predictive modelling, clinician oversight, and patient education is therefore crucial to maintaining trust and sustaining positive attitudes toward adoption. As emphasised in previous studies, ethically governed personalisation can strengthen therapeutic alliance without compromising user autonomy (Blease & Torous, 2023; Yu et al., 2023).

### Augmenting clinical practice and supporting professional capacity

Experts in this study consistently emphasised that GAI should be viewed as a tool that enhances, rather than replaces, clinical expertise. This reflects a strong sense of perceived usefulness, as experts recognised GAI’s potential to automate routine tasks, support clinical reasoning, and improve workflow efficiency. Participants noted that GAI can summarise clinical sessions, highlight risk indicators, and provide rapid insights for triage and assessment. As E10 remarked, *“AI does in seconds what takes clinicians hours—summarising sessions, detecting patterns, and even suggesting actions. That gives us more time, actually, to listen.”* These findings align with research indicating that AI can improve diagnostic accuracy and streamline administrative processes, enabling clinicians to devote more attention to direct patient care (Rollwage et al., 2024; Sendak et al., 2020).

Experts also discussed the perceived ease of use of GAI tools, particularly their ability to integrate seamlessly into existing digital-health systems. E9 observed, *“Unlike humans, AI doesn’t get tired. It assists in real-time, cross-checking diagnoses, prompting questions, and reducing documentation fatigue.”* Such integration enhances clinicians’ willingness to adopt GAI, particularly in low-resource settings where mental health professionals face substantial caseloads and documentation burdens (Cheng et al., 2023; Weiner, 2022). Moreover, by automating repetitive tasks, GAI may help reduce burnout and improve the overall quality of professional life (Hamdoun et al., 2023).

Nonetheless, participants expressed reservations closely tied to trust and professional accountability. As E3 cautioned, *“Ethical review must be ongoing. Clinicians must remain accountable. AI is a tool, not a decision-maker.”* This aligns with the ethical position that GAI should complement but never replace human judgement in mental-health contexts (Blease & Torous, 2023; Torous & Blease, 2024). Although clinicians generally expressed positive attitudes toward GAI’s potential to enhance efficiency and accuracy, they stressed that technological adoption must remain subordinate to human oversight and empathy (Balcombe, 2023; Miner et al., 2020). Future research should therefore explore collaborative clinician–AI frameworks that integrate ethical governance, accountability, and professional responsibility as core design principles (Allen & Woodnutt, 2023; Choudhury et al., 2025).

### Transforming therapeutic modalities and experiences

Experts described GAI as an emerging and evolving tool capable of transforming the way therapeutic experiences are delivered and supported. This reflects a combination of perceived usefulness and attitude toward use, as experts viewed GAI as an innovative complement to traditional therapeutic modalities. Current applications include AI-guided journaling, conversational rehearsal, and simulated exposure exercises that supplement or, in some cases, substitute for therapist-led interventions. E7 explained, *“Clients used ChatGPT to rehearse conversations, explore thoughts, and reflect on emotional triggers. It became part of their therapeutic toolkit.”* These insights align with evidence indicating that conversational AI systems can facilitate reflection, emotional regulation, and self-guided cognitive restructuring between sessions (Balcombe, 2023; Liu et al., 2023).

Looking ahead, participants anticipated that GAI would contribute to more emotionally intelligent and culturally adaptive therapeutic environments. As E9 observed, *“These systems are becoming companions. They remember what you’ve said, respond in emotionally intelligent ways, and build trust over time.”* This perception underscores the importance of trust, a critical extension of the Technology Acceptance Model in mental health contexts (Blease & Torous, 2023). When users perceive GAI systems as empathetic, reliable, and contextually responsive, their emotional engagement and therapeutic continuity increase. These findings align with the growing literature that emphasises the importance of empathetic AI responses in strengthening user trust and therapeutic alliance when ethically guided (Elyoseph et al., 2023; Howcroft et al., 2025).

Nonetheless, scepticism persists regarding the authenticity of synthetic empathy and the limitations of machine-mediated understanding. Some experts questioned whether emotionally responsive GAI could truly replicate the depth of human attunement. As E9 reflected, *“Predicting emotional states is one thing, but feeling them is another.”* This tension highlights that acceptance of GAI in therapy depends not only on perceived usefulness but also on concerns about relational authenticity, ethical sensitivity, and the preservation of human connection (Doraiswamy et al., 2020; Ettman & Galea, 2023). Addressing these challenges requires interdisciplinary collaboration among affective computing, psychology, and ethics to ensure that emotionally intelligent systems enhance, rather than diminish, the therapeutic alliance (Allen & Woodnutt, 2023).

### Leveraging collective intelligence for research and public health

Beyond individual clinical care, experts identified the collective potential of GAI to advance mental health research and inform public health strategies. This reflects a strong sense of perceived usefulness, as participants emphasised that GAI can synthesise data from thousands of interactions to detect population-level patterns in mood, stress, and emotional well-being. As E13 noted, *“AI can analyse thousands of conversations and tell us what’s changing—mood trends, anxiety spikes, sleep patterns. It’s population-level mental health in real time.”* These insights align with frameworks in learning health systems and precision public health that leverage data-driven approaches to improve intervention design (Elyoseph et al., 2023; Friedman et al., 2015; Xian, 2024).

Experts also highlighted the role of GAI in mental health education and public engagement, noting that its scalability makes it an effective medium for promoting mental health literacy and reducing stigma (Liu et al., 2023; Sharma et al., 2023). By delivering psychoeducational content and empathetic dialogue, GAI can normalise mental health discussions and encourage early help-seeking behaviour (Balcombe, 2023). Such applications foster positive attitudes toward use among both professionals and the public, positioning GAI as a credible adjunct to community mental health promotion.

Despite these benefits, experts repeatedly underscored the importance of trust in large-scale data utilisation. As E14 warned, *“There’s no legal framework for data protection in many regions. We risk harming under the guise of innovation.”* This concern underscores that user trust and data governance are inextricably linked to successful adoption. Without robust privacy safeguards, transparent algorithms, and culturally sensitive regulatory frameworks, perceptions of GAI’s usefulness may deteriorate (Blease & Torous, 2023; Choudhury et al., 2025). Thus, the responsible expansion of GAI in public health must be accompanied by ethical data governance that ensures safety, accountability, and social responsibility (Allen & Woodnutt, 2023; Balaskas et al., 2025).

### Ethical governance and global accountability

Although experts did not identify ethical considerations as the most immediate benefit of GAI, they consistently emerged as essential for the sustainable adoption of GAI. The key issues raised included algorithmic bias, opacity in decision-making processes, and the lack of enforceable regulatory frameworks. As E5 observed, *“AI systems are only as ethical as their inputs and governance. Right now, both are inadequate.”* This reinforces the argument that technological advancement must be matched by ethical innovation and governance reform (Allen & Woodnutt, 2023; Doraiswamy et al., 2020).

From the perspective of the Technology Acceptance Model, these findings underscore trust as both a prerequisite and determinant of adoption. Experts emphasised that regardless of how useful or easy to use GAI may appear, its acceptance in mental health care depends heavily on confidence in its integrity, transparency, and accountability (Blease & Torous, 2023; Torous & Blease, 2024). A lack of trust arising from biased algorithms or opaque data practices can undermine perceived usefulness and erode professional attitudes toward adoption. Therefore, fostering trust through ethical design, explainability, and continuous oversight is critical for sustaining long-term acceptance among clinicians and patients (Balaskas et al., 2025; Choudhury et al., 2025).

Experts further emphasised the need for global accountability and context-sensitive regulation to ensure equitable and culturally appropriate implementation. As E14 stated, *“We’re operating in a global system without global standards. What’s ethical in one context may be harmful in another.”* This underscores the importance of flexible governance systems that respect cultural diversity in ethical interpretation, privacy norms, and mental health practices. Collaborative policymaking among clinicians, technologists, ethicists, and community stakeholders is thus vital to establish shared standards of safety and inclusivity (Elyoseph et al., 2023; Moran, 2024).

While developments such as the ISO/IEC 42001 standard for AI lifecycle management represent progress, experts agreed these frameworks remain insufficient in isolation. There is an urgent need to integrate ethical design principles throughout the entire AI lifecycle from data collection and algorithm training to deployment and evaluation (Allen & Woodnutt, 2023). Embedding ethics at every stage will help ensure that GAI tools enhance rather than exacerbate existing inequities.

In sum, the experts’ perspectives reaffirm that ethical governance is inseparable from the acceptance of GAI in mental health care. Strengthening transparency, accountability, and cultural sensitivity can reinforce trust, shape positive professional attitudes, and ultimately support the responsible global adoption of GAI systems (Blease & Torous, 2023; Doraiswamy et al., 2020; Torous & Blease, 2024).

## Conclusion

The results of this Delphi study suggest that generative GAI is regarded by mental health professionals as offering meaningful benefits for the sector, particularly in enhancing accessibility, supporting personalised interventions, and strengthening professional capacity. Across both rounds, experts consistently positioned GAI as the most promising when used as a collaborative and informative tool, ranking this direction above concerns about technological or computational constraints. These priorities reflect how perceived usefulness, trust, and favourable professional attitudes shape expectations surrounding the integration of GAI into mental health practice.

Acknowledging the limitations of this study is important. The interpretation of the findings relies heavily on the expertise and judgement of the participating professionals (Schmalz et al., 2021). Although the Delphi method draws strength from informed consensus, expert perspectives may vary across disciplines, cultural backgrounds, and experience, thereby introducing an element of subjectivity. Furthermore, the panel comprising psychiatrists, clinical lecturers, medical doctors, counsellors, and researchers may not fully capture the breadth of perspectives found across the wider mental health workforce or the general public.

To enhance representativeness, future studies should broaden panel composition to include participants from public, private, and non-governmental sectors. Diversifying expertise would reduce individual bias, enrich professional insight, and improve the generalisability of findings across varied service contexts.

Methodologically, this study demonstrates the value of combining qualitative thematic analysis, Delphi techniques, and quantitative validation to achieve triangulation. This mixed-methods design strengthened the credibility of the findings by integrating professional insight with measurable consensus, though it also introduced challenges related to data integration and sustained participant engagement. Future researchers may refine this design to maintain methodological rigour while improving efficiency.

Beyond methodological considerations, further research is required to explore how GAI shapes mental health outcomes more broadly. Important areas of enquiry include patterns of use, the quality of human–AI interaction, and the social, organisational, and individual factors that influence its effects. Longitudinal studies are essential for understanding the long-term implications for patient well-being, while comparative research can help identify which tools best serve diverse populations and clinical settings.

From a policy perspective, responsible implementation of GAI must address data privacy, algorithmic bias, informed consent, and equitable access. Regulatory frameworks, transparency standards, and digital inclusion initiatives will be crucial for ensuring that GAI technologies are deployed ethically and sustainably.

By aligning innovation with fairness, professional responsibility, and human-centred values, GAI can contribute meaningfully to a more accessible and responsive mental health care ecosystem. Continued collaboration among clinicians, researchers, and policymakers will be critical to realising this potential while safeguarding ethical standards and public trust.

## Data Availability

Data supporting the findings of this study are not publicly available because of confidentiality agreements and ethical restrictions. However, the data may be made available by the corresponding author upon reasonable request. The metadata record of the dataset was active and archived at https://zenodo.org/records/15117187.
